# Efficacy of Anlotinib for the Treatment of Angiosarcoma of the Face and Neck: A Case Report

**DOI:** 10.3389/fonc.2021.596732

**Published:** 2021-06-28

**Authors:** Biyong Ren, Wei Wang, Jing Tan, Bo Yuan, Guilan Chen, Xiaofei Mo, Jieqiong Fan, Bo Yang, Xiaoping Huang

**Affiliations:** ^1^ Department of Medical Oncology, Chongqing University Three Gorges Hospital, Chongqing, China; ^2^ Department of Gastrointestinal, Thyroid and Vascular Surgery, Chongqing University, Three Gorges Hospital, Chongqing, China; ^3^ Department of Oncology, Yunyang County People’s Hospital, Chongqing, China

**Keywords:** anlotinib, angiosarcoma of face and neck, case report, partial response, progression-free survival

## Abstract

Angiosarcoma of the face and neck is a rare soft tissue sarcoma with a high degree of malignancy. The current treatment methods mainly rely on a combination of surgery and radiotherapy and/or chemotherapy. However, the options for drug treatment are very limited and surgery can be difficult to carry out due to the location of the tumor, so the efficacy of first-line drugs needs to be constantly explored. A case of angiosarcoma of the head and face diagnosed by biopsy is reported here. The patient received an oral anlotinib hydrochloride capsule once a day (12 mg on days 1 - 14/1 week off for a 21-day cycle) due to the difficulty of surgery. Until now (April, 2020), after 10 months of treatment, the patient’s scalp and facial lesions have gradually reduced and the partial response and progression-free survival of this patient were good, with moderate or tolerable adverse events. This approach provides a new approach for the clinical treatment of malignant angiosarcoma of the face and neck with anlotinib as first-line therapy.

## Introduction

Angiosarcoma (AS) is a rare soft tissue sarcoma (STS) that arises from the endothelial cells of blood vessels or the lymphatics and accounts for about 2% of all soft tissue sarcomas (1–4). AS can occur in all parts of the body and is a malignant sarcoma with high metastasis and recurrence rates. In particular, AS of the head and face is the most common type, accounting for about 60% of cases (2), with a lower survival and higher recurrence rate (5). Currently, the treatment of AS relies on the comprehensive treatment mode of surgical resection, radiotherapy and chemotherapy, while the choice of targeted therapy is very limited, and the treatment effect is often unsatisfactory, with only a 10~50% 5-year survival rate (6, 7). The growth and metastasis of tumor cells depends on the formation of new blood vessels. Vascular endothelial growth factor (VEGF) is the most effective tumor angiogenesis stimulant. VEGF binds to its receptors (VEGFR), causing a series of signal transductions, the release of multiple cytokines and stimulation of vascular (lymphatic) endothelial cell proliferation and migration. AS is derived from vascular endothelial cells, and it has been reported that VEGF protein and VEGFR are overexpressed in 80% of AS patients (8–11). Therefore, VEGF may be an important factor in the survival, growth, progression and metastasis of this type of tumor.

Anlotinib is a recombinant humanized monoclonal IgG1 antibody of VEGF. It is a competitive antagonist of VEGF and blocks its biological activity, reducing existing tumor vascular degeneration and inhibition of the growth of new blood vessels. It also has an anti-permeability effect on tumor blood vessels, facilitating the ability of chemotherapy drugs to reach tumor tissues or cells more easily, ultimately inhibiting tumor growth and exerting an anti-tumor effect (12). In a phase 2 study, anlotinib showed anti-tumor activity in several STSs that progressed after anthracycline-based chemotherapy (13). The current report is a case study of an elderly patient with AS of the scalp who received anlotinib therapy in our hospital after signing informed consent.

## Case Description

The complaints of the patient (a 99 year old male) was that he had patchy edema erythema on the left side of his head since May 2019. Subsequently, the erythema area gradually expanded without exudation and was occasionally itchy. At the same time, mild edema developed in both eyes and the erythema further spread to the head. During this period (> 2 months), the patient went to several local hospitals and received treatment such as external medication, but without any improvement in his symptoms. The erythema further spread to the left side of his face.

**Figure 1:**
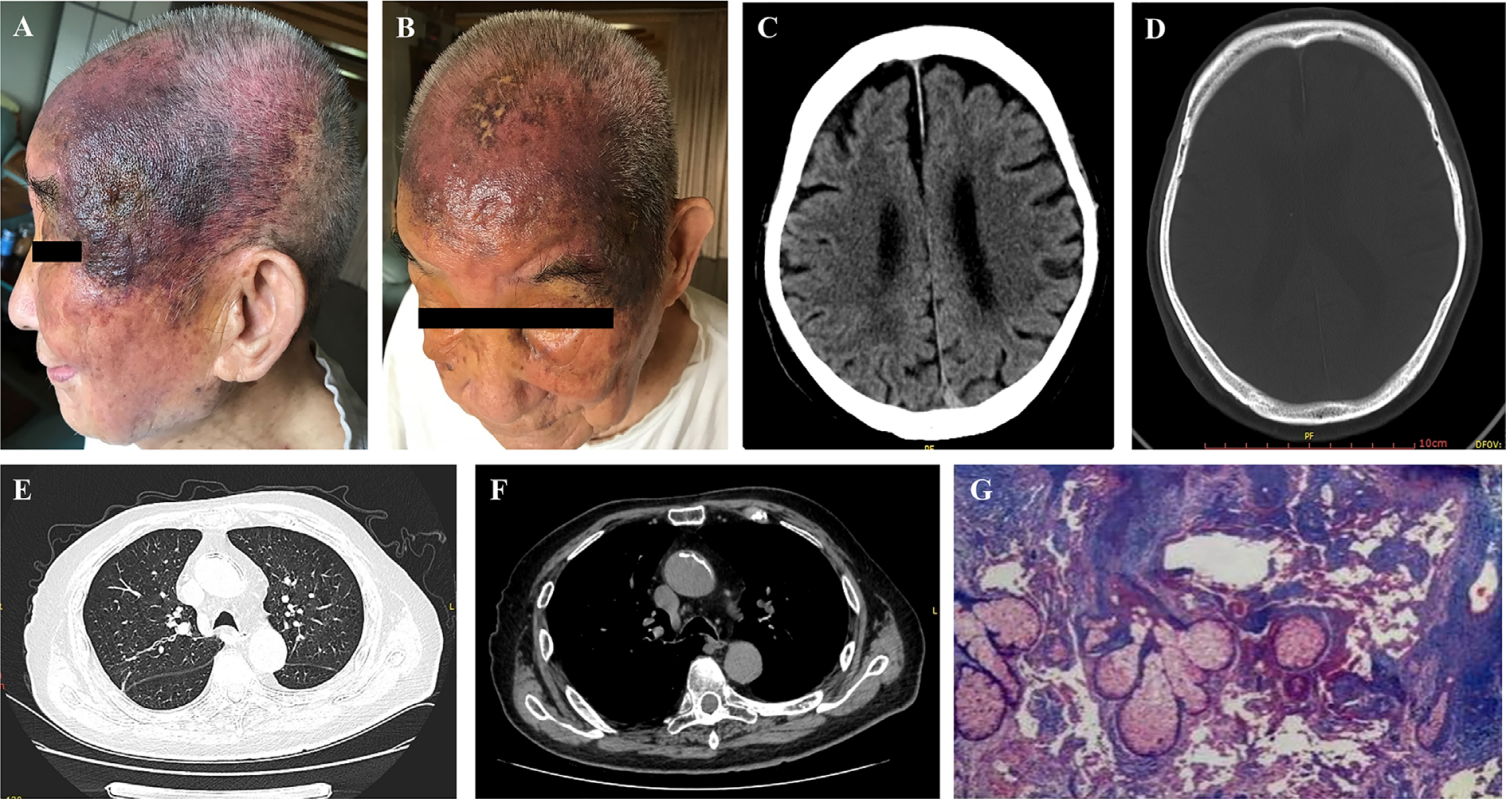
Examination before treatment including dermatological inspection **(A, B)**, head CT **(C, D)**, chest CT **(E, F)** and pathological assessment **(G)**.

A medical examination was conducted and revealed no abnormalities. Dermatological examination showed clear dark red or purplish red infiltrating patches on the head. They did not completely fade after pressing, with visible exudation and scabs on the surface. The surface of the infiltrating erythema of the left face showed densely distributed vesicles with a little exudation. Mild edema was observed around both eyes, as shown in **Figures 1A, B**.

A head CT scan was performed on May 28^th^ 2019 at the People’s Hospital of Yunyang County. The scan revealed atrophy and a widened bilateral frontal subdural space. Liver, kidney and blood coagulation functions were all normal (**Figures 1C, D**). A chest CT performed on June 5^th^ 2019 showed that a few fiber proliferation foci were scattered in the right lung and the patient had a gallstone and also aortic sclerosis. The bilateral pleura were thickened and adhered. Cysts may have been present in the left lobe of the liver and in the right kidney (**Figures 1E, F**). Therefore, we made further examinations in the dermatology department of Chongqing Southwest Hospital on July 8, 2019. Samples from the left temporal lobe were obtained and clinically diagnosed. A pathological assay revealed that the epidermis was slightly hypertrophic. A number of fissured blood vessel formations were observed as well as vascular endothelial cell proliferation that was considered to be mild atypia. We also found a large degree of extravasation of red blood cells, and dermal inflammatory cell infiltration to varying degrees. The laboratory measurements revealed CD31 (+), CD34 (+), ERG (+), Ki67:50% (+), S100 (-), and CK (-) (**Table 1**), which were pathologically confirmed as AS (**Figure 1G**). The other indicators of routine blood tests are presented in **Table 1**. Combining all the data together, this patient was diagnosed with senile AS of the head and face and was referred to our oncology department for appropriate treatment.

**Table 1:** Results of laboratory measurements.

	Before treatment	After treatment
CD31	+	
CD34	+	
ERG	+	
Ki67	50.00% (+)	
S100	–	
CK	–	
WBC (/L)	5.78 × 10^9^	5.55 × 10^9^
RBC (/L)	4.55 × 10^12^	4.87 × 10^12^
Hb (g/L)	140.00	146.00
PLT (/L)	177.00 × 10^9^	183.00 × 10^9^
Occult blood		Negative
C-Reactive protein		Negative
Total bilirubin (µmol/L)		20.07
Direct bilirubin (µmol/L)		6.90 (0 - 6.8)
Indirect bilirubin (µmol/L)		13.17
Total protein (g/L)		67.00
Albumin (g/L)		37.20
Globulin (g/L)		29.80
A/G		1.25 (1.25 - 2.5)
Glutamic-pyruvic transaminase (U/L)		9.20
Glutamic oxalacetic transaminase (U/L)		14.67
Alkaline phosphatase (U/L)		68.00
Glutamyltranspeptidase (U/L)		90.00
Ureophil (mmo1/L)		5.37 (2.86 - 8.20)
Creatinine (µmol/L)		105.58 (21.5 - 104)
Uric Acid (µmol/L)		403.25 (208 - 428)
Prothrombin time (s)		11.30 (9 - 14)
Mobility (%)		97.30 (70 - 150)
International normalized ratio (PT)		0.97 (0.8 - 1.5)
Fibrinogen (g/L)		4.31 (2 - 4)
Activated partial thromboplastin time		26.80 (20 - 40)
Thrombin time (s)		16.00 (14 - 21)
D-Dimer (mg/L)		1.77 (0 - 0.55)

## Therapeutic Interventions and Outcomes

**Figure 2:**
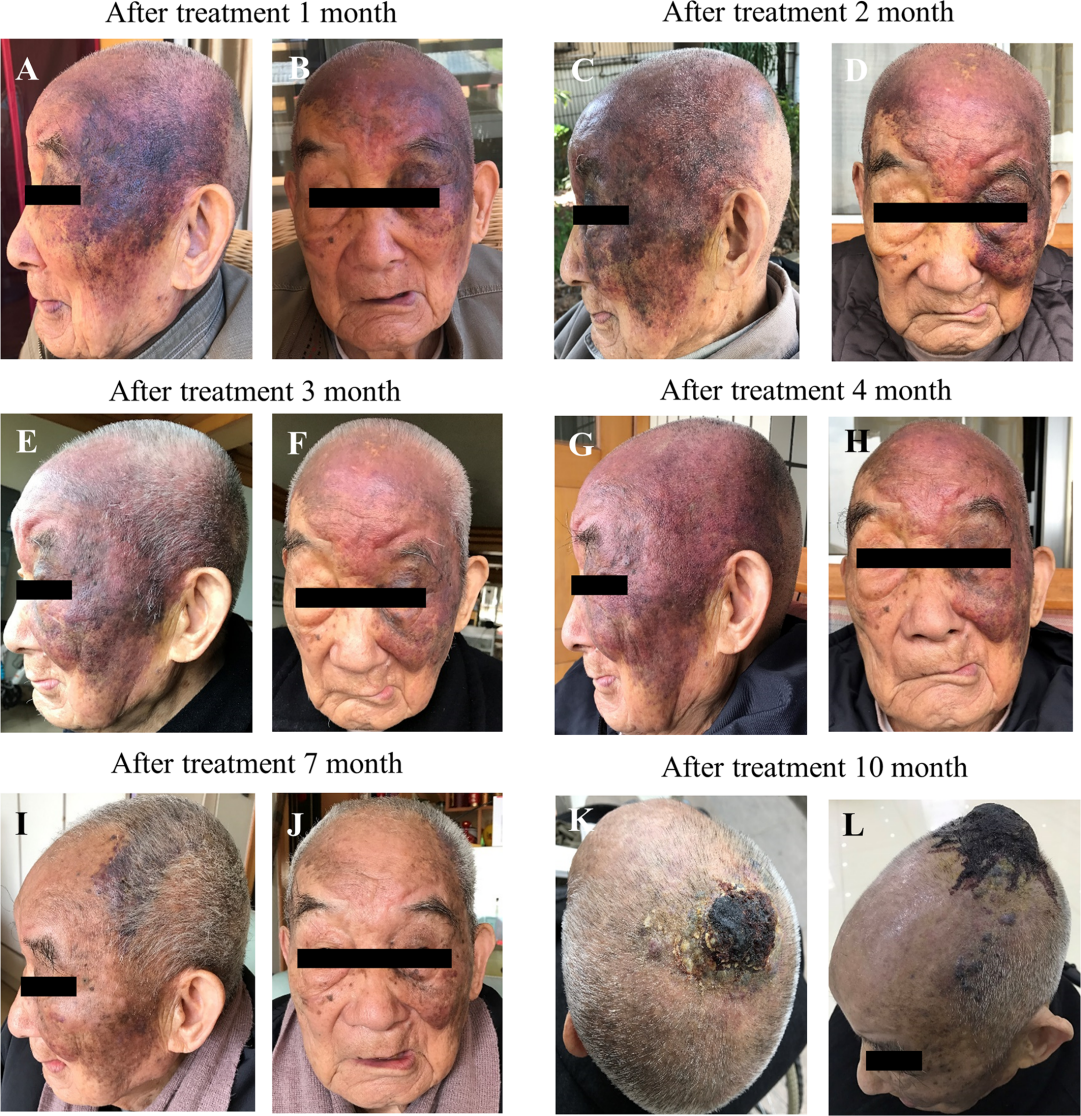
Effect of anlotinib on scalp and facial lesion changes over a course of 10 months treatment. **(A–J)** left side lateral view and right side frontal view, **(K–L)** superior views.

The patient started to take anlotinib hydrochloride from July 20 2019, with 12 mg tablets once a day, orally, d1-14, and then rest for 7 days, as 1 cycle. If the patient suffered a loss of appetite or the patient’s diet was cut by more than half or blood pressure was elevated with dizziness symptoms, anlotinib hydrochloride was administered once every other day, 12 mg tablets orally each time. According to the Common Terminology Criteria for Adverse Events (CTCAE) (14) grading, the patient suffered from fatigue (grade 1), which was treated by rest, reduced appetite (grade 3), treated with orally and intravenously administered nutritional supplements and elevated blood pressure (133-189/72-107 mmHg) (grade 2-3) during the period of medication. Candesartan tablets were used to treat high blood pressure. The patient returned to a normal dose after the relief of symptoms. The clinical examinations showed that the scalp and facial lesions were gradually controlled, after an initial increase during the 1^st^ month as shown in **Figure 2**. The current treatment efficacy was evaluated as a partial response (PR) according to the Response Evaluation Criteria in Solid Tumors (RECIST 1.1), since more than a 30% decrease in the sum of the longest diameters of the target lesions compared to the baseline sum of the longest diameters was detected. Progression-free survival (PFS) was over 10 months, with tolerable adverse reactions.

**Figure 3:**
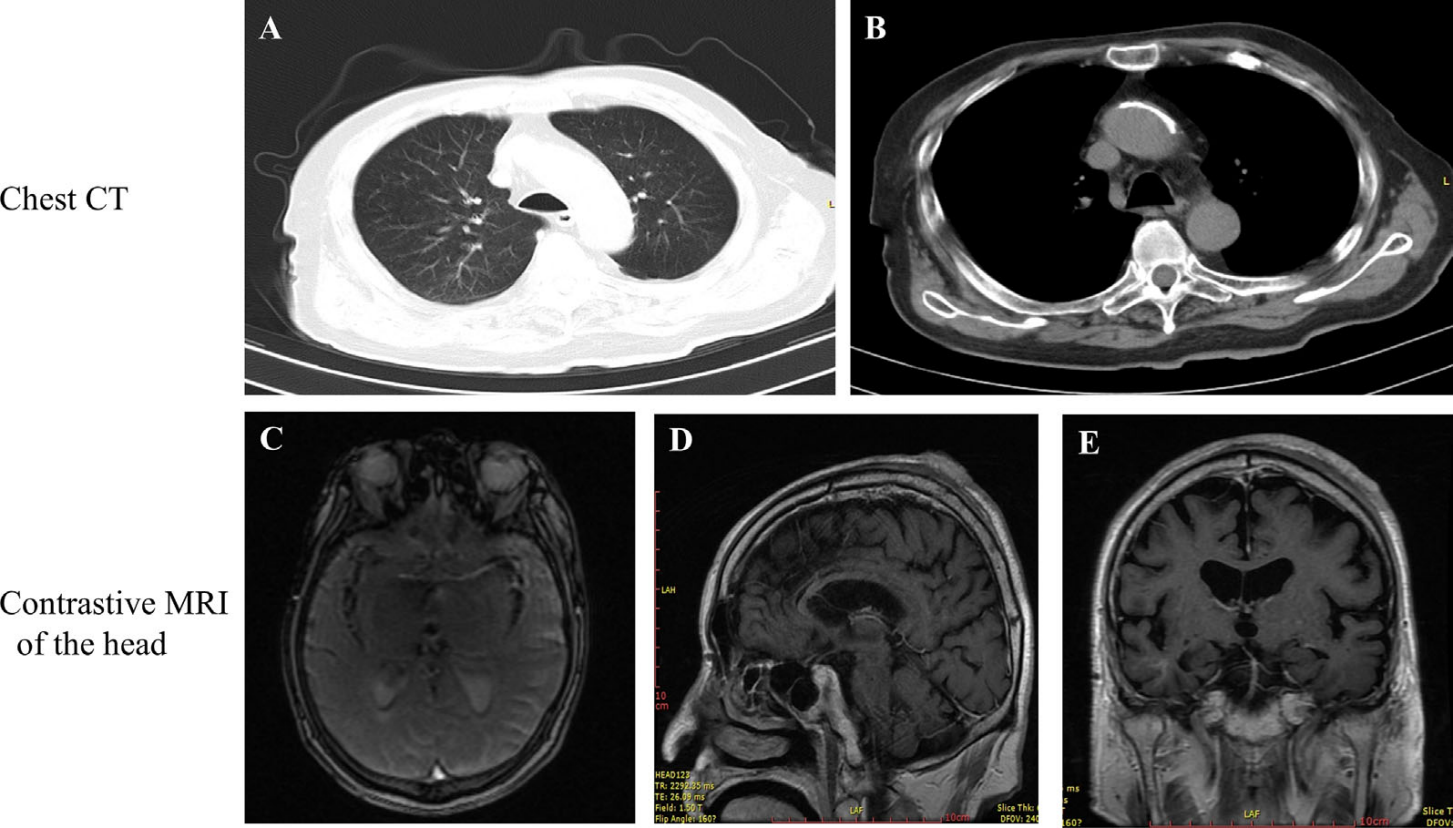
**(A, B)** Examination of chest CT. **(C)** superior view, **(D)** lateral view and **(E)** posterior view of the scull during contrast MRI after 10 months treatment with anlotinib.

On March 18^th^, 2020, the patient was examined in the People’s Hospital of Yunyang County, and all indicators were normal (**Table 1**). A chest CT revealed a low number of fiber proliferation foci scattered in the right lung; local thickening and adhesion of the bilateral pleura; right diaphragmatic surface swelling; aortic sclerosis; possible cysts in the left lobe of the liver and right kidney; gallbladder stones; bone hyperplasia of the thoracic vertebra and compressibility changes in the lumbar vertebra (**Figures 3A, B**). Contrast MRI of the head after diagnosis revealed multiple abnormal spots and patchy signals in the center of the bilateral semiovale and under the prefrontal parietal cortex. T2WI exhibited a high signal with a clear boundary, TIFLAIR presented a slightly lower signal and T2FLAIR exhibited no high signal. No abnormal signs were found after intracranial enhancement. A high signal was recorded in the T2 flair strips of the deep white matter surrounding the bilateral lateral ventricles. The bilateral subdural space was widened and bilateral lateral ventricle symmetry was slightly enlarged. The cerebral sulcus was marginally widened but without a significant shift in the midline structure. The soft tissue shadow at the top of the head was thickened and enhanced (**Figures 3C–E**). The diagnosis results were as follows: 1) Ischemia foci in the bilateral hemi oval center and frontal parietal lobe; 2) Demyelinating changes of white matter and brain atrophy; and 3) Soft tissue swelling at the top of the head with visible enhancement. All these AS related findings indicated the efficacy of anlotinib as first-line treatment, but more observations will be necessary before reaching a definitive conclusion.

## Discussion

The case was an elderly male patient with extensive AS of the head and face. Chemotherapy is widely used to treat metastatic cutaneous angiosarcoma (cAS) and for lesions that cannot be completely resected. In different studies, doxorubicin-based regiments were considered to be the gold standard for STS. This case is a 99-year-old elderly patient with extensive lesions, who was not deemed suitable for surgery. For radiotherapy the range of the lesion was too great and doxorubicin therapy has a number of side effects including cardiac and blood reactions. In addition, the patient and his relatives refused chemotherapy and therefore anlotinib was selected as his first-line treatment. The results showed that the scalp and facial lesions were gradually controlled and evaluated as PR, with a PFS of 10 months up to now. Adverse reactions grades 1-3 occurred during treatment, but did not lead to discontinuation of anlotinib medication. AS is a rare STS with low survival and high recurrence rates. The standard treatment for cAS patients is surgical resection, with or without postoperative radiotherapy. A review of 764 case records revealed that surgery alone, rather than radiation or chemotherapy, was associated with improved survival (15). Compared with other therapies, the 5-year survival rate was improved by surgery [odds ratio (OR) ¼ 4.369; P ¼ 0.002] (16). Positive resection margins were associated with poor overall survival (OS) (17). Patients with cAS should always be considered for radical surgery. Although there are no clear guidelines for the specific width of the surgical margin of cAS, a margin of ≥ 3 cm and a deep margin are strongly recommended (18).

Surgery remains the preferred treatment for patients with AS of the scalp, but due to its rapid development, patients may develop diffuse or generalized local cancer and therefore are not suitable for subsequent surgery. Surgery combined with radiotherapy provides the best OS time and is the most commonly used treatment for this highly aggressive tumor (19, 20). A study at the University of Texas MD Anderson Cancer Center showed that patients who received combination therapy had a statistically higher OS rate than those who received radiation or surgery alone (21). Postoperative radiotherapy can improve the survival rate (5, 22, 23), and high dose radiation (> 50 Gy) and extended treatment are generally recommended. The median survival time of patients who received radiotherapy was 4 times that of patients who did not receive radiotherapy (*P* = 0.033). Patients who received combined surgery and radiotherapy had a better prognosis than those who received surgery or radiotherapy alone (HR = 0.16, *P* = 0.006) (22). Ogawa et al. retrospectively analyzed 48 patients with localized cephalic and facial angiosarcoma, and found that the 2-year OS rates of patients who underwent surgery combined with radiotherapy, surgery or radiotherapy, and no treatment were 45.8%, 11.1%, and 0%, respectively, with the best prognosis being for patients who received surgery combined with radiotherapy (*P* < 0.000) (24).

Chemotherapy is widely used to treat metastatic cAS and lesions that cannot be completely resected (25). Current data of chemotherapy regimens for cAS are inconsistent. While a number of studies have shown the benefits of adjuvant chemotherapy, others have reported no increase in overall survival (21, 26, 27). Doxorubicin-based regiments were considered to be the gold standard treatment for STS, with PFS being about 3 months (28, 29). However, taxanes (paclitaxel) have recently been found to be more effective than doxorubicin, with an average PFS of 4-5 months (28–30). Paclitaxel was rigorously evaluated in a phase 2 trial in which 30 patients with unresectable or metastatic AS were recruited (30). The results showed that the 2-month and 4-month progression-free rates were 74% and 42%, respectively.

Tyrosine kinase inhibitors (e.g., pazopanib, sorafenib, acitinib) are used for the treatment of various STSs, including cAS. Studies that have evaluated pazopanib have produced conflicting results. Kolla et al. retrospectively evaluated 40 patients with AS treated with pazopanib. Compared with other STSs, pazopanib showed a similar efficacy for the treatment of AS, with a median PFS and OS of 3 and 9.9 months, respectively (31). The PALETTE study was a large phase 3 study that recruited 372 STS patients with different histological subtypes, and unequivocally demonstrated the clinical efficacy of pazopanib (32). However, the PALETTE study did not address whether pazopanib was effective for the treatment of cAS. A study in Japan reported that cAS patients treated with pazopanib had a PFS of 94 days (33), which was in line with those reported in the PALETTE study, implying the usefulness of pazopanib therapy for cAS. However, a report that analyzed 8 cAS patients treated with pazopanib showed no statistically significant difference in OS between cAS patients who received or did not receive pazopanib (34). Sorafenib alone was effective only in patients with AS who had received chemotherapy, with an overall response rate of 23% after 2 months of treatment and 0% for patients who had not received chemotherapy (35). The VEGFR inhibitor, bevacizumab, has been investigated as monotherapy and as combination therapy. Considering its anti-angiogenic properties, bevacizumab should effectively treat cAS. In 2 patients with nasal angiosarcoma, both experienced a complete response with bevacizumab treatment, which is the first publicly reported treatment of AS with bevacizumab (36). A multicenter phase 2 clinical trial enrolled 23 AS patients, who were treated with bevacizumab for 6 weeks. Two patients achieved partial remission and 11 were stable, with a mean PFS of 12 weeks (37). The trial demonstrated that bevacizumab, as a monotherapy, was effective in treating AS with an average progression time of 26 weeks. A phase 2 clinical trial found that compared with paclitaxel alone, combined paclitaxel and bevacizumab did not increase the clinical benefit for patients with AS. Two groups of patients had a median PFS of 6.6 months, while the median overall survival was 19.5 months and 15.9 months, respectively (38).

Anlotinib is a novel tyrosine kinase inhibitor that targets a variety of factors involved in tumor proliferation, vascular system angiogenesis and the tumor microenvironment (39). Anlotinib inhibits VEGF/VEGFR signaling by selectively targeting VEGFR-2, -3, and FGFR-1, -2, -3, and -4 with high affinity. Anlotinib also inhibits the activity of PDGFR, C-Kit, Ret, aurora B, C-FMS and the discoidin domain receptor 1 (DDR1), thereby significantly inhibiting the proliferation of tumors (39). In preclinical studies, anlotinib exhibited extensive anti-tumor activity in a variety of xenograft models (39). A phase 2b clinical trial that used anlotinib hydrochloride capsules for the treatment of advanced STS found that it had good efficacy and safety in these patients (40). To evaluate anlotinib in recurrent metastatic STS, a phase 2 clinical study involving 166 patients was carried out. The results showed that the overall 12-week PFS rate and ORR rate were 68% and 13% respectively, with overall readily controllable adverse events (13). On June 24^th^ 2019, the NMPA approved anlotinib treatment for adenosine STSs, clear cell sarcomas and other advanced STSs, which had progressed or relapsed after at least prior treatments with anthracycline chemotherapy.

Cutaneous angiosarcoma is a tumor that originates from vascular endothelial cells and has highly expressed VEGF and VEGFR. Hence, anlotinib was administered at 12 mg, po, qod for the first 2 weeks and off 1 week for a 21-day cycle. The results showed that the scalp and facial lesions were gradually controlled and evaluated as PR with a PFS of 10 months. The adverse reactions to treatment were mild and tolerable. The follow-up of this patient is ongoing.

## Conclusions

Vascular-targeted drugs may be of great clinical value for the treatment of scalp AS. For inoperable patients, vascular targeted drugs like anlotinib may bring new hope to patients with AS of the scalp. Its long-term efficacy needs to be further studied.

## Data Availability Statement

The datasets used and/or analyzed during the current study are available from the corresponding author on reasonable request.

## Ethics Statement

Written informed consent was obtained from the individual for the publication of any potentially identifiable images or data included in this article.

## Author Contributions

BR was responsible for the conception and design of the study. BR, WW, JT, BYu, and GC collected the data. BR and WW were in charge of statistical analysis. BR, WW, and BYu were responsible for analysis and interpretation of the data. BR drafted the manuscript. BR, WW, XM, and JF revised and commented on the draft. All authors contributed to the article and approved the submitted version.

## Conflict of Interest

The authors declare that the research was conducted in the absence of any commercial or financial relationships that could be construed as a potential conflict of interest.
